# Recent Progress in the Nanoscale Evaluation of Piezoelectric and Ferroelectric Properties via Scanning Probe Microscopy

**DOI:** 10.1002/advs.201901391

**Published:** 2020-07-29

**Authors:** Owoong Kwon, Daehee Seol, Huimin Qiao, Yunseok Kim

**Affiliations:** ^1^ School of Advanced Materials and Engineering & Research Center for Advanced Materials Technology Sungkyunkwan University (SKKU) Suwon 16419 Republic of Korea

**Keywords:** conductive atomic force microscopy, ferroelectricity, piezoelectricity, piezoresponse force microscopy, scanning probe microscopy

## Abstract

Piezoelectric and ferroelectric materials have garnered significant interest owing to their excellent physical properties and multiple potential applications. Accordingly, the need for evaluating piezoelectric and ferroelectric properties has also increased. The piezoelectric and ferroelectric properties are evaluated macroscopically using laser interferometers and polarization–electric field loop measurements. However, as the research focus is shifted from bulk to nanosized materials, scanning probe microscopy (SPM) techniques have been suggested as an alternative approach for evaluating piezoelectric and ferroelectric properties. In this Progress Report, the recent progress on the nanoscale evaluation of piezoelectric and ferroelectric properties of diverse materials using SPM‐based methods is summarized. Among the SPM techniques, the focus is on recent studies that are related to piezoresponse force microscopy and conductive atomic force microscopy; further, the utilization of these two modes to understand piezoelectric and ferroelectric properties at the nanoscale level is discussed. This work can provide guidelines for evaluating the piezoelectric and ferroelectric properties of materials based on SPM techniques.

## Graphical abstract

Piezoelectric and ferroelectric materials have been of significant interest owing to their remarkable physical properties. This Progress Report summarizes the recent advances in the nanoscale evaluation of piezoelectric and ferroelectric properties of diverse materials using approaches based on scanning probe microscopy.

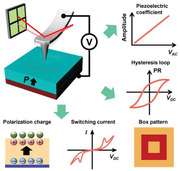

## 1 Introduction

In the past several decades, piezoelectric and ferroelectric materials have attracted significant interest owing to their excellent physical properties and wide range of potential applications in information technology,^[^ ^1^, ^2^

^]^ sensors/actuators,^[^ ^3^, ^4^, ^5^ ^]^ and energy harvesting devices.^[^ ^6^, ^7^, ^8^ ^]^ Accordingly, the evaluation of piezoelectric and ferroelectric properties has also become important. Laser interferometry has been employed for the evaluation of macroscopic piezoelectric properties; in this technique, the voltage‐induced surface displacement generated by the converse piezoelectric effect is monitored.^[^ ^9^ ^]^ Furthermore, polarization–electric field (*P*–*E*) hysteresis loops can be measured to macroscopically evaluate the polarization charge density under the application of DC triangular waveforms.^[^ ^10^, ^11^, ^12^, ^13^, ^14^ ^]^ In addition, second harmonic generation and polarized light microscopy^[^ ^15^ ^]^ have been used to examine inversion symmetry breaking, that is, the presence of polarization,^[^ ^16^ ^]^ and macroscopic domain structures,^[^ ^17^ ^]^ respectively. In these methods, the diffraction and polarization of light waves in response to the piezoelectric and ferroelectric properties of materials are measured.

However, while macroscopic tools have been widely used to explore macroscopic piezoelectric and ferroelectric properties, the application of these techniques can sometimes be limited; this is because, with respect to sizes and dimensions, the research focus has shifted from bulk crystals and ceramics to thin films, nanostructures,^[^ ^18^, ^19^, ^20^, ^21^ ^]^ and 2D materials.^[^ ^22^, ^23^, ^24^ ^]^ For instance, the preparation of electrodes for measuring the properties of nanodots or similar nanostructures is nearly impossible. Furthermore, *P*–*E* hysteresis loops can be distorted when leakage currents of the measured sample, that is, ultra‐thin films, are relatively high, because their measurement is based on the detection of current. In addition, the spatial resolution of the optical‐based method is limited to the macro‐ and microscopic scales because of the diffraction limit and optical wavelength of the instruments.^[^ ^16^, ^25^, ^26^ ^]^ In fact, in the measurement of the *P*–*E* hysteresis loop, spatially resolved switching information cannot be obtained; this is because the obtained switching information is gathered over the entire area of a capacitor, that is, it is based on the averaged information of the capacitor. These limitations can be a significant hurdle for obtaining spatially varied information at small scales. Simultaneously, the types of materials studied have expanded from oxides to transition metal dichalcogenides (TMDs),^[^ ^22^, ^23^, ^24^ ^]^ organics,^[^ ^27^, ^28^ ^]^ biological materials,^[^ ^29^, ^30^, ^31^, ^32^, ^33^ ^]^ and hybrid materials.^[^ ^34^, ^35^, ^36^ ^]^ Because some of these materials exhibit relatively weaker piezoelectric and ferroelectric properties, their properties cannot be easily observed using macroscopic techniques.

Meanwhile, scanning probe microscopy (SPM) techniques have been suggested as an alternative approach for evaluating the piezoelectric and ferroelectric properties at the nanoscale level. Furthermore, SPM techniques can enhance the existing technologies for measuring signals that cannot be monitored via macroscopic techniques. For example, in macroscopic *P*–*E* hysteresis loop measurements, the measured area is confined to the top electrode; the diameter of this electrode is usually at least a couple of micrometers. However, if this is combined with SPM, a *P*–*E* hysteresis loop can be obtained over less than one micrometer for the top electrode; furthermore, it is possible to measure the *P*–*E* hysteresis loop on the film surface without the top electrode.^[^ ^37^, ^38^, ^39^ ^]^

In this progress report, we summarize recent achievements in the evaluation of piezoelectric and ferroelectric properties on the nanoscale based on SPM techniques. Among the various SPM methods presented in **Table** **1**, we first discuss the recent progress and some issues such as quantification methods and non‐piezoelectric contributions made in piezoresponse force microscopy (PFM); this is because PFM is the most well‐known SPM technique for elucidating the piezoelectric and ferroelectric properties. Subsequently, we shift our focus on recent studies related to current detection‐based SPM modes and discuss the utilization of these modes to understand piezoelectric and ferroelectric properties at the nanoscale further.

**Table 1:** SPM techniques for evaluating piezoelectric and ferroelectric properties

Property	Method (abbreviation^a)^)		Applications
Piezoelectricity	PFM	*V*_ac_ amplitude sweep	Piezoelectric nonlinearity^[^ ^114^, ^130^ ^]^ and piezoelectric coefficient measured by the converse piezoelectric effect.^[^ ^41^, ^104^, ^113^ ^]^
	DPFM		Piezoelectric coefficient measured by the direct piezoelectric effect.^[^ ^164^ ^]^
	Nano‐indentation		Piezoelectric coefficient measured by the converse piezoelectric effect.^[^ ^23^ ^]^
Ferroelectricity	PFM	Box patterns	Domain switching.^[^ ^19^, ^117^, ^121^, ^123^, ^126^ ^]^
		PFM spectroscopy	Domain switching and switching dynamics.^[^ ^43^, ^113^, ^132^, ^133^, ^136^ ^]^
	cKPFM spectroscopy		Discrimination of ferroelectricity.^[^ ^177^ ^]^
	DPFM		Polarization charge density.^[^ ^164^ ^]^
	G–IV		Polarization charge density, capacitance, and dielectric constant.^[^ ^39^ ^]^
	AFM‐PUND		Polarization charge density.^[^ ^38^ ^]^

a) Abbreviations: PFM (piezoresponse force microscopy); DPFM (direct piezoelectric force microscopy); cKPFM (contact Kelvin probe force microscopy); G–IV (general mode I–V); and AFM‐PUND (positive‐up‐negative‐down atomic force microscopy).

## 2 PFM

### 2.1 Recent Advance in PFM

**Figure 1:**
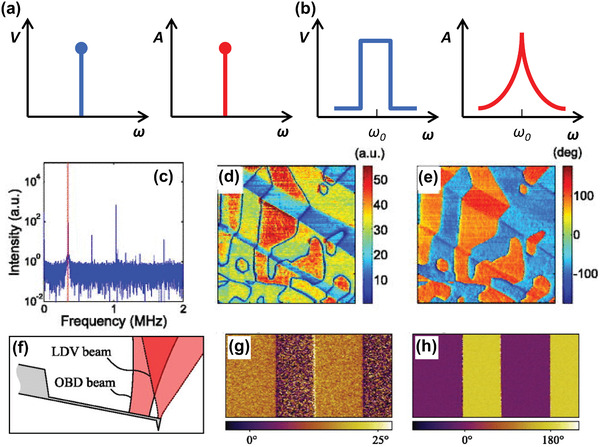
Operational principle of PFM: a) single‐frequency and b) BE methods. c) Overall, the G‐PFM response with a red line shows the location of the first mode at 345 kHz. G‐PFM d) amplitude and e) phase at 345 kHz. (c–e) were reproduced with permission.^[^ ^50^ ^]^ Copyright 2015, AIP Publishing LLC. f) Schematic of the side view of the optical paths for the LDV and OBD beams focused on the cantilever. g) OBD and h) LDV measurements of the phase over domains in a PPLN reference sample. (f–h) were reproduced with permission.^[^ ^51^ ^]^ Copyright 2015, AIP Publishing LLC.

Since its development, PFM has been extensively utilized to explore nanoscale piezoelectric and ferroelectric properties in diverse material systems.^[^ ^40^, ^41^, ^42^, ^43^, ^44^, ^45^ ^]^ In PFM, an AC voltage (*V* *_ac_*) with a specific frequency is applied to the conductive SPM tip to induce surface displacement, that is, an electromechanical response, by the converse piezoelectric effect.^[^ ^46^ ^]^ Conventionally, single‐frequency far below the first contact resonance frequency (CRF) has been used for performing PFM measurements (**Figure** **1**a). In general, the obtained PFM amplitude and phase signals can provide information on the magnitude of the piezoresponse and the polarization direction, respectively. Nevertheless, for achieving higher signal‐to‐noise ratio and more accurate measurement, PFM has been developed in terms of the operational program and component advancements.

Although single‐frequency excitation has been generally used for performing PFM measurements, the band excitation (BE) technique, as an example of the operational program advancements, can be used to improve the detection resolution and the signal‐to‐noise ratio, for example, pm level of out‐of‐plane (OP) resolution. Because BE can track the CRF between the SPM cantilever and sample surface by applying an AC waveform with a band frequency, as shown in Figure 1b,^[^ ^47^, ^48^, ^49^ ^]^ the response amplified by the BE technique can provide a better signal‐to‐noise ratio. While the BE technique is only operated in the contact resonance regime, general mode PFM (G‐PFM), which allows the acquisition of the cantilever deflection over a full frequency range, was recently developed by Kalinin and co‐workers.^[^ ^50^ ^]^ In G‐PFM, data can be separately analyzed at a specific frequency after the acquisition of the complete cantilever response. For instance, the G‐PFM amplitude and phase at 345 kHz were visualized by separate analyses of the G‐PFM response, as shown in Figure 1c–e. By simultaneously capturing multimodal cantilever responses, such as OP and in‐plane (IP) deflection, multidimensional information can be obtained, and piezoelectric and ferroelectric properties can be analyzed.

Furthermore, component development has also contributed to improving accuracy of the PFM measurements. In addition to the conventional optical beam deflection (OBD) method, PFM measurements using a laser Doppler vibrometer (LDV) instead of OBD have been reported by Proksch and co‐workers (Figure 1f).^[^ ^51^ ^]^ The advantage of the LDV method is that its sensitivity is intrinsic, and therefore, it can provide a more quantitative value. The cantilever response is affected by undesirable electrostatic forces, which can affect the PFM signal measured by the OBD method (see the location of the laser spot in Figure 1f). However, as long as the laser spot of the LDV method is directly above the cantilever tip, as shown in Figure 1f, the LDV method is less sensitive to electrostatic effects compared to the conventional OBD method.^[^ ^51^ ^]^ Therefore, in periodically poled lithium niobate (PPLN), the PFM phase measured by the LDV method shows the expected 180° phase shift between domains, unlike the PFM phase measured by the OBD method (Figure 1g,h). Thus, this can be an alternative method for obtaining more quantitative information compared to the conventional PFM discussed in Section 2.2. Furthermore, Dharmasena et al.^[^ ^52^ ^]^ reported that the decoupling between topography and PFM signal was possible by a new design of cantilever, for example, inner‐paddled cantilever, instead of conventional microcantilevers. This indicates that changing of cantilever design^[^ ^52^, ^53^, ^54^, ^55^ ^]^ could improve the PFM measurements.

### 2.2 Quantification of the PFM Amplitude Signal

In PFM, the magnitude of surface displacement, which is referred to as the PFM amplitude, is induced by the converse piezoelectric effect. Fundamentally, the magnitude of the OP surface displacement, which is referred to as the OP‐PFM amplitude, can be quantified by employing three methods: reference‐sample‐, static‐sensitivity‐, and dynamic‐sensitivity‐based quantifications.

In the reference‐sample‐based method, the OP‐PFM amplitude can be quantified by comparing the OP‐PFM amplitude measured for the specific sample with that of standard reference samples.^[^ ^56^, ^57^ ^]^ However, this method requires an appropriate reference sample with well‐defined geometry in terms of domain orientation, thickness, topography, and so on.^[^ ^58^, ^59^ ^]^ Furthermore, the physical properties of the reference sample, such as the symmetry group and piezoelectric tensor, should be considered because they might affect the PFM amplitude signal.^[^ ^60^ ^]^ Thus, if the reference sample is not well‐defined, the quantified OP‐PFM amplitude can be inaccurate.

The static‐sensitivity‐based quantification method uses the force–distance (*F*–*D*) curve for measuring the static sensitivity, that is, the inverse optical lever sensitivity (InvOLS).^[^ ^61^, ^62^, ^63^ ^]^ In the *F*–*D* curve, static sensitivity in units of nm V^−1^ can be obtained by calculating the reciprocal slope of the *F*–*D* curve. In general, because the OP‐PFM amplitude detected by the OBD is expressed as a voltage unit, the representation in units of distance can be obtained by multiplying this quantification factor by the measured the OP‐PFM amplitude signal.

However, since the static shape of a vibrating cantilever is not identical to its dynamic shape above the first CRF,^[^ ^51^, ^61^ ^]^ static sensitivity can generally be used when the OP‐PFM amplitude is measured at far below the first CRF. Thus, to improve the accuracy of quantification while considering the dynamic shape of the cantilever, the dynamic‐sensitivity‐based method was recently suggested by Balke et al.^[^ ^63^ ^]^ For describing the dynamic sensitivity of a cantilever that is in close proximity to the first CRF, the dynamic shape factor is calculated by comprehensively considering the cantilever shape, contact stiffness, Q‐factor, and CRF via theoretical simulations. More recently, Killgore et al. suggested an experimental approach based on an interferometer to acquire the dynamic shape factor experimentally without using analytical models.^[^ ^64^ ^]^ Based on this, the dynamic sensitivity is defined as the static sensitivity divided by the dynamic shape factor and is used to calibrate the OP‐PFM amplitude signal, which is measured near the first CRF. Each quantification method has its own advantages and disadvantages for quantifying the OP‐PFM amplitude, as briefly summarized in **Table** **2**.

**Table 2:** Advantages and disadvantages of quantification methods for OP‐PFM amplitude signal

Quantification method	Advantage	Disadvantage
Reference sample^[^ ^57^, ^75^ ^]^	Relatively easy to quantify.	A reference sample with well‐defined structures, such as orientation and thickness, is required.
Static sensitivity^[^ ^51^ ^]^	No additional samples required. Easy to measure *F*–*D* curves.	Usually limited far below the first contact resonance frequency.
Dynamic sensitivity^[^ ^63^, ^64^ ^]^	Reflects dynamic shape of the cantilever near the contact resonance frequency.	Additional theoretical or experimental processes are necessitated to calculate the dynamic shape factor.

These indicate that the physical parameters of the material, such as the effective piezoelectric coefficient *d*_33,eff_, obtained via the OP‐PFM amplitude, can be quantified. However, the OP‐PFM exhibits intrinsic limitations for achieving the same level of quantitative values as those measured macroscopically using top electrodes.^[^ ^65^, ^66^ ^]^ These limitations include the non‐uniform electric field distribution underneath the SPM tip,^[^ ^66^ ^]^ complex cantilever dynamics,^[^ ^61^, ^63^ ^]^ instrumental background noise,^[^ ^67^, ^68^ ^]^ and electrostatic artifacts generated between the SPM cantilever/tip and the sample (see also Section 2.3.).^[^ ^69^, ^70^ ^]^ Furthermore, the measurements can be influenced by the clamping effect.^[^ ^71^, ^72^ ^]^ Accordingly, the values obtained by OP‐PFM cannot fundamentally provide the same quantitative value to those measured by macroscopic techniques using top electrodes.^[^ ^65^, ^66^, ^73^ ^]^ Nonetheless, if such drawbacks can be properly considered,^[^ ^51^, ^63^, ^67^, ^74^ ^]^ similar values of the effective piezoelectric coefficient can be obtained using PFM.^[^ ^65^ ^]^ Thus, the quantification of the measured OP‐PFM amplitude can still provide insights into physically significant information regarding the piezoelectricity at the nanoscale.

Meanwhile, in addition to the quantification of the OP‐PFM amplitude, the quantification of the IP‐PFM amplitude has been reported.^[^ ^75^, ^76^, ^77^ ^]^ By utilizing the damping effect of friction force, Choi et al. obtained the scan distance dependent lateral torsional signal curve by tracking the lateral signal from both trace and retrace scans, thus extracting the lateral InvOLS, in unit of nm V^−1^, for the quantification of IP‐PFM amplitude.^[^ ^76^ ^]^ This method was further improved by Wang et al. with better sensitivity. The magnitude and direction of the local shear displacement can be quantitatively measured through quantifying the buckling and torsional motion using a calibrated AFM scanner tube.^[^ ^77^ ^]^ However, this method is limited to the application of the complicated cases, for example, materials with both OP and IP polarizations.

Aside from abovementioned method, Lei et al. suggested a reference‐sample‐based method for simultaneous OP‐PFM and IP‐PFM quantification.^[^ ^78^ ^]^ The optical amplification factor of a cantilever can be determined by PFM imaging across the 180˚ domain wall on a reference sample, then the factor can be used for the quantification of the targeted sample by scanning across the *c*/*a* domain wall using the same cantilever. However, because this quantification method provides effective piezoelectric coefficients containing elastic and dielectric information, finite element method should be used to understand the effective coefficients in terms of intrinsic piezoelectric tensor coefficients.

In addition, it was reported experimentally and theoretically that the shear piezoelectricity could lead to unexpected IP‐PFM signal at 180˚ domain walls in purely *c*‐axis oriented ferroelectrics due to breaking down of the crystal symmetry induced by polarization switching.^[^ ^79^, ^80^, ^81^ ^]^ Although there is still controversy on the dominant driving mechanism,^[^ ^82^, ^83^ ^]^ the IP‐PFM signal should be carefully interpreted in these cases. Furthermore, the IP‐PFM differs from OP‐PFM in some aspects, for instance, 1) while the IP‐PFM signal decays dramatically at a specific frequency due to the onset of sliding friction between tip and sample surface, the OP‐PFM signal is nearly independent of frequency far below resonance frequency;^[^ ^69^, ^84^, ^85^ ^]^ 2) with increasing an AC voltage, the OP‐PFM amplitude will increase linearly, but the IP‐PFM amplitude can saturate due to the onset of sliding friction.^[^ ^86^, ^87^ ^]^ Overall, although there have been several attempts for the quantification of the IP‐PFM amplitude, it is relatively less explored compared to those on the quantification of the OP‐PFM amplitude. Further studies might be necessary to examine the quantification methods for the IP‐PFM amplitude.

### 2.3 Non‐Piezoelectric Contributions to the PFM Signal

**Figure 2:**
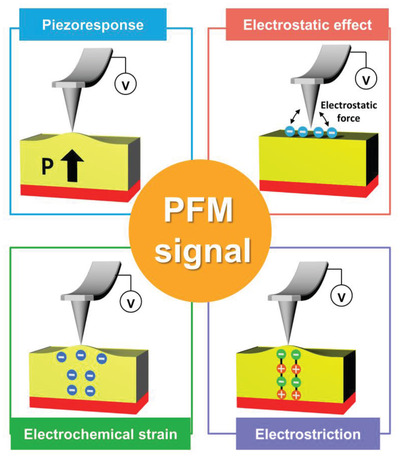
Schematic of the contributions of several phenomena to the PFM signal: piezoresponse, electrostatic effect, electrochemical strain (Vegard strain), and electrostriction; *P* indicates polarization, and the colored circles represent ions (or surface charges).

Although PFM has been widely used for exploring piezoelectric and ferroelectric properties at the nanoscale, it has been recently considered that the PFM hysteresis loop can also originate from non‐piezoelectric contributions, such as electrostatic effects and electrochemical strain, as shown in **Figure** **2**.^[^ ^88^, ^89^ ^]^ The electrostatic effect originates from the Coulombic electrostatic force between the SPM cantilever/tip and the sample surface and is usually an inevitable interaction in PFM as well as in other voltage‐modulated SPM modes.^[^ ^90^, ^91^, ^92^ ^]^ It is challenging to remove these effects from the PFM signal entirely. For example, the electrostatic effect caused by the surface potential, that is, the contact potential difference, can result in the misinterpretation of the polarization direction in PFM images^[^ ^70^, ^93^ ^]^ and can further induce phase flipping in the hysteresis loops notwithstanding the absence of actual switching.^[^ ^94^, ^95^ ^]^ Moreover, the electrostatic effect caused by charge injection could change the surface potential from the equilibrium state.^[^ ^95^, ^96^, ^97^, ^98^, ^99^ ^]^ Consequently, ferroelectric‐like PFM image contrast and hysteresis loop can be induced by the electrostatic effect associated with the charge injection. Nonetheless, several previous studies have demonstrated how to minimize the electrostatic force, for example, increasing the spring constant of the SPM cantilever,^[^ ^41^, ^100^ ^]^ conducting measurements at higher AC frequencies^[^ ^101^ ^]^ and compensating the surface potential by applying an external voltage.^[^ ^70^, ^92^ ^]^

In the case of electrochemical strain, surface displacement occurs underneath the SPM tip owing to the Vegard strain in ionically active materials.^[^ ^102^, ^103^, ^104^, ^105^ ^]^ A local surface volume change can be caused by local ion redistribution induced by external electric fields underneath the SPM tip. Therefore, ferroelectric‐like hysteresis loops can be formed in non‐ferroelectric materials, such as LiCoO_2_ and lithium‐ion conductors.^[^ ^104^, ^105^, ^106^, ^107^, ^108^ ^]^ These results revealed that it is likely to misinterpret the ionically mediated PFM hysteresis loop as ferroelectricity. Accordingly, several studies have distinguished these aspects from each other based on voltage conditions, harmonic responses, and environmental conditions.^[^ ^36^, ^96^, ^104^, ^109^ ^]^

Overall, apart from electrostatic effects and electrochemical strain, there can be other potential non‐piezoelectric contributions,^[^ ^91^, ^109^, ^110^, ^111^, ^112^ ^]^ including electrostriction, Joule heating, and flexoelectricity. Because the complete decoupling of the undesired non‐piezoelectric contributions from the PFM signal is not straightforward, careful interpretation of the PFM signal may be important for verifying the presence of piezoelectricity and ferroelectricity using PFM.^[^ ^88^, ^89^ ^]^ Therefore, in addition to PFM, in this progress report, we introduce other methods based on SPM techniques to evaluate the piezoelectric and ferroelectric properties.

## 3 Evaluation of Piezoelectricity and Ferroelectricity Using PFM

### 3.1 Evaluation of Piezoelectricity

**Figure 3:**
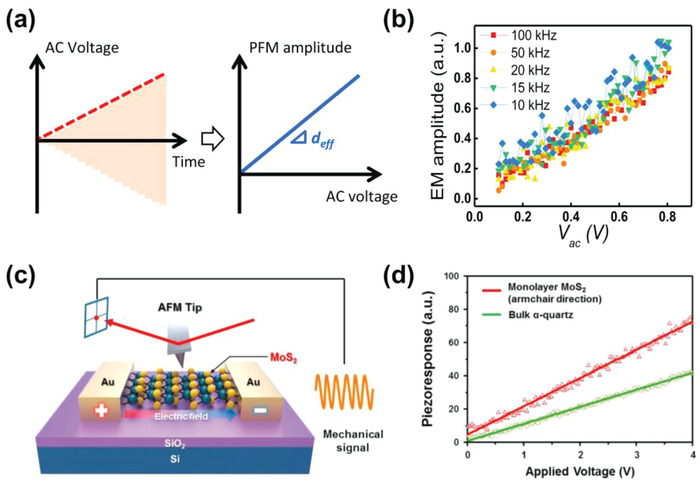
a) Plot of the voltage waveform of *V*_ac_ amplitude sweep (left) and the PFM amplitude versus *V*_ac_ amplitude (right). b) *V*_ac_ amplitude sweep results for PZT thin films. (b) was reproduced with permission.^[^ ^104^ ^]^ Copyright 2016, Springer Customer Service Center GmbH: Springer Nature. c) Schematic of the measurement configuration for IP‐PFM on monolayer MoS_2_. d) IP piezoresponse as a function of the electric field applied in a monolayer MoS_2_ flake through two lateral electrodes. The solid lines represent the fits by a linear equation. (c) and (d) were reproduced with permission.^[^ ^75^ ^]^ Copyright 2016, Elsevier.

In general, the effective piezoelectric coefficient can be measured by monitoring the PFM amplitude with respect to the magnitude of the *V*_ac_ amplitude in PFM, which is referred to as the *V*_ac_ amplitude sweep.^[^ ^41^, ^62^, ^113^, ^114^ ^]^ This approach can be implemented by applying a gradually increasing *V*_ac_ amplitude (**Figure** **3**a) to the SPM tip. Subsequently, the PFM amplitude is be varied by gradually increasing the *V*_ac_ amplitude and plotted against the magnitude of the *V*_ac_ amplitude. Because piezoelectricity exhibits a linear relationship between mechanical strain and electric field, the linear slope of the plot of the PFM amplitude versus the *V*_ac_ amplitude corresponds to the effective piezoelectric coefficient.

Figure 3b shows the representative results of the OP piezoelectricity, for example*, d*_33,eff_, measured by the *V*_ac_ amplitude sweep in ferroelectric thin films. Seol et al.^[^ ^104^ ^]^ revealed an evident linear relationship between the PFM amplitude and the applied voltage in a classical ferroelectric Pb(Zr,Ti)O_3_ (PZT) thin film and further showed that the OP‐PFM amplitude is not significantly changed in the kilohertz frequency range. If there is an apparent linear relationship between the PFM amplitude and applied voltage, the slope, that is, the effective piezoelectric coefficient, of the measured results can be extracted by linear fitting. However, because there can be non‐piezoelectric contributions to the PFM signal, as discussed in Section 2.3., the linear relationship between the PFM amplitude and applied voltage can be observed without the existence of OP piezoelectricity. Thus, the PFM signal should be carefully interpreted to prove the existence of the OP piezoelectricity.

In addition to the OP piezoelectric measurement, the IP piezoelectricity, for example*, d*_11,eff_, can be measured by applying the *V*_ac_ amplitude sweep through the lateral electrodes and analyzing the corresponding IP piezoresponse. Figure 3c shows a schematic of the IP‐PFM setup for measuring the IP piezoelectric coefficient, *d*_11,eff_, of a monolayer MoS_2_ device. Among 2D materials, TMDs such as MoS_2_ and MoTe_2_ possess IP piezoelectricity owing to their IP inversion symmetric breaking.^[^ ^22^, ^115^, ^116^ ^]^ Based on the measurement configuration shown in Figure 3c, Kim et al.^[^ ^75^ ^]^ reported the IP piezoelectricity of chemical vapor deposition‐grown monolayer MoS_2_ compared with the piezoelectricity of quartz, as shown in Figure 3d. However, although IP piezoelectricity was measured in monolayer MoS_2_ through observation of the IP‐PFM signal, the effect of the substrate on the intrinsic piezoelectricity and the non‐piezoelectric contributions to the measured IP‐PFM signal were not completely eliminated. Furthermore, because IP‐PFM measurements can be affected by various factors, such as the excitation frequency, cantilever stiffness, and tip torsion/sliding, the approach is still limited in quantitative evaluation. Accordingly, further studies should be performed to obtain quantitative IP‐PFM measurements, as discussed in Section 2.2.

### 3.2 Evaluation of Ferroelectricity

PFM has also been used to explore the ferroelectric behavior of diverse systems, such as oxides, polymers, biological materials, and 2D materials, at the nanoscale. In PFM, two main approaches are considered for characterizing ferroelectricity, namely the imaging and spectroscopy modes.

**Figure 4:**
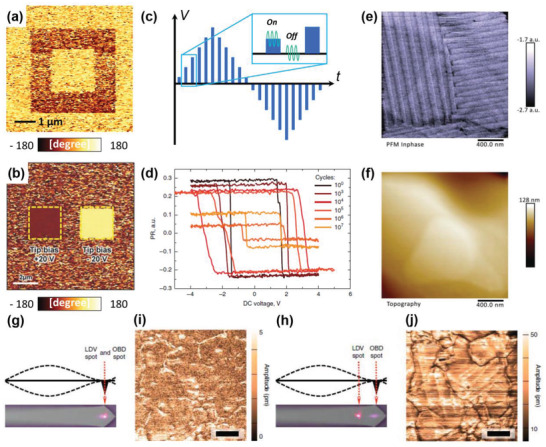
Ferroelectric domain imaging after poling in a) BFO and b) P(VDF‐TrFE). (a) and (b) were reproduced with permission.^[^ ^121^, ^122^ ^]^ Copyright 2018 and 2016, Wiley‐VCH Verlag GmbH & Co. KGaA, respectively. c) Schematic of the pulse‐type triangular waveform for PFM hysteresis loop measurement. d) PFM hysteresis loop for PZT nanocapacitors. Figure (d) was reproduced with permission.^[^ ^131^ ^]^ Copyright 2019, Springer Customer Service Center GmbH: Springer Nature. e,f) PFM phase and topography images of a typical MAPbI_3_ sample surface, respectively. (e) and (f) were reproduced with permission.^[^ ^141^ ^]^ Copyright 2017, The Royal Society of Chemistry. g,h) LDV–PFM measurements at a drive frequency of 335 kHz. The side‐view illustrations of the expected mode shape of the cantilever show decoupling (g) and coupling (h) of the signal with cantilever dynamics; the bottom insets show top‐view images of the laser spot location on the cantilever. i,j) Amplitude images with the LDV spot in the locations (g) and (h), respectively. Scale bars, 1 µm. (g–j) were reproduced with permission.^[^ ^162^ ^]^ Copyright 2018, Springer Customer Service Center GmbH: Springer Nature.

In ferroelectric materials, the direction of spontaneous polarization, that is, electrical dipole, can be switched by applying an external voltage (or field), that is, a poling procedure. Poling can also be performed with SPM because the SPM tip can be used as a movable top electrode for applying a voltage to the sample. Thus, in addition to the examination of domain structures, when combined with the poling procedure, the PFM imaging mode can be utilized to examine the switching behavior.^[^ ^117^, ^118^, ^119^, ^120^ ^]^ **Figure** **4**a,b show the PFM phase images after the poling procedures with different box patterns in BiFeO_3_ (BFO) and P(VDF‐TrFE) thin films, respectively.^[^ ^121^, ^122^ ^]^ A 180° phase difference can be observed in the PFM phase images when both upward and downward polarizations occur. In addition, if pulses can be applied with different pulse widths and voltages without scanning the surface (instead of box patterns), a more detailed switching behavior under different pulse conditions can be explored.^[^ ^19^, ^110^, ^119^, ^120^, ^123^, ^124^, ^125^, ^126^, ^127^, ^128^, ^129^ ^]^

The advantage of the PFM imaging mode is that the local domain structure and domain motion can be easily visualized. Thus, if similar measurements are conducted in ferroelectric capacitors as a function of the pulse conditions, it is feasible to explore the switching dynamics in the structure of the ferroelectric capacitor using PFM by analyzing the switched area.^[^ ^18^, ^19^, ^123^, ^127^, ^129^, ^130^ ^]^ In general, it is not physically straightforward for the PFM imaging mode combined with the poling procedure to provide complete information on switching parameters, such as the coercive voltage and magnitude of the remnant piezoresponse; this is because the process requires extensive scanning at different pulse conditions. Such information can be conveniently explored with the PFM spectroscopy mode.

The spectroscopic approaches, such as PFM hysteresis loop measurements, have been widely used to explore ferroelectric behaviors. The PFM hysteresis loop is typically measured at a fixed point by applying a pulse‐type triangular voltage waveform, as shown in Figure 4c. The hysteresis loop can be obtained by applying a probing *V*_ac_ while the DC voltage is either applied (on‐field) or not applied (off‐field). We note that the PFM signal is generally obtained in the off‐field state to minimize the electrostatic contribution.^[^ ^93^ ^]^ Figure 4d shows the representative PFM hysteresis loop as a function of the number of switching cycles measured in the PZT nanocapacitors.^[^ ^131^ ^]^ As shown in the figure, the hysteresis loop clearly shows a rectangular shape, indicating ferroelectric polarization switching. This hysteresis loop measurement can be further extended for exploring spatially varying local switching behaviors based on grid‐type measurements (i.e., spectroscopy), such as switching spectroscopy‐PFM (SS‐PFM),^[^ ^130^, ^132^, ^133^, ^134^, ^135^ ^]^ SS‐PFM based on the first‐order reversal curve (FORC) waveform, referred to as FORC‐SS‐PFM,^[^ ^20^, ^133^ ^]^ and switching dynamics spectroscopy‐PFM.^[^ ^136^ ^]^

In addition to conventional ferroelectric materials, organic–inorganic hybrid perovskite (OIHP) materials, for example, methyl ammonium lead iodide (MAPbI_3_),^[^ ^34^, ^36^, ^137^, ^138^, ^139^, ^140^, ^141^, ^142^, ^143^ ^]^ which have been typically studied for solar‐cell applications owing to their anomalous photovoltaic effect, have been recently studied by PFM.^[^ ^35^, ^144^, ^145^, ^146^, ^147^ ^]^ Numerous studies have investigated the presence of ferroelectricity in OIHP materials using PFM;^[^ ^36^, ^148^, ^149^, ^150^, ^151^, ^152^ ^]^ this is because their relatively higher photo‐conversion efficiency as well as current–voltage (*I*–*V*) hysteresis can be associated with ferroelectricity owing to their perovskite crystal structures.^[^ ^153^, ^154^, ^155^, ^156^ ^]^ For instance, Kutes et al.^[^ ^152^ ^]^ reported as‐grown ferroelectric domain structures in MAPbI_3_ and their switching by an external electric field. However, although some PFM studies have demonstrated the presence of ferroelectricity in OIHP materials, the topic is still controversial.^[^ ^137^, ^141^, ^142^, ^157^, ^158^, ^159^, ^160^, ^161^ ^]^ For instance, Xiao et al.^[^ ^157^ ^]^ reported that a PFM hysteresis loop was not observed for MAPbI_3_. Furthermore, with respect to the twin domain structures of MAPbI_3_, Rohm et al.^[^ ^141^, ^161^ ^]^ presented the ferroelectric nature of the domains by using PFM (Figure 4e,f), while, using LDV–PFM combined with other techniques, Liu et al. showed that the twin domain structures originate from the effects of chemical segregation and elastic variation rather than ferroelectricity (Figure 4g–j).^[^ ^162^, ^163^ ^]^ This disagreement can be caused not only by the complexity of this class of materials but also by the presence of the non‐piezoelectric contributions in PFM signals, as discussed in Section 2.3.^[^ ^88^, ^89^ ^]^ Because it is challenging to decouple the non‐piezoelectric properties completely and the properties of interest in the PFM signal, careful interpretation of PFM imaging as well as spectroscopy is required to understand the ferroelectric properties of materials exactly. Therefore, it is worth introducing other SPM techniques to evaluate the piezoelectricity and ferroelectricity, as discussed in the following sections.

## 4 Evaluation of Piezoelectricity and Ferroelectricity Using Other SPM‐Based Techniques

### 4.1 Evaluation of Piezoelectricity

**Figure 5:**
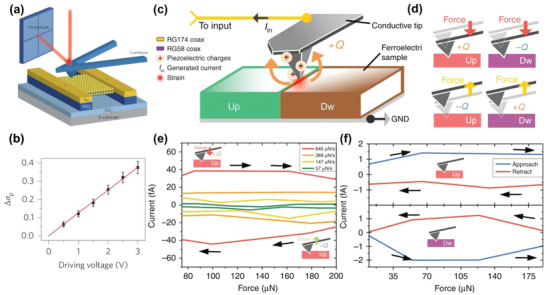
a) Schematic of the process of probing the piezoelectric properties of freestanding monolayer MoS_2_. For measuring the in‐plane piezoelectric stress, the MoS_2_ film was suspended on two hydrogen silsesquioxane posts and clamped underneath by two Au electrodes; b) corresponding piezoelectric stress measured at various driving voltages at a fixed depth of indentation. (a) and (b) were reproduced by permission.^[^ ^23^ ^]^ Copyright 2015, Springer Customer Service Center GmbH: Springer Nature. c) Schematic of the DPFM setup. d) Schematic of the piezoelectric charge generation in the case of applying and releasing strain for the force–distance spectroscopy measurements. e) Force/time rate applied on the tip and f) the aligned direction of domain‐dependent piezoelectric charge generation in force–distance spectroscopy. (c–f) were reproduced with permission.^[^ ^164^ ^]^ Copyright 2017, Springer Customer Service Center GmbH: Springer Nature.

In addition to conventional PFM measurements, other methods for exploring piezoelectric properties using SPM techniques have been reported recently. Zhu et al. demonstrated the measurement of piezoelectricity based on the converse piezoelectric effect in freestanding monolayer MoS_2_.^[^ ^23^ ^]^ In their experimental setup, freestanding monolayer MoS_2_ was used to prevent substrate effects on the intrinsic piezoelectric properties; subsequently, the SPM tip was used to measure the piezoelectric properties of the freestanding monolayer MoS_2_, as presented in **Figure** **5**a. They measured the converse piezoelectric effect, that is, the piezoelectric stress induced by the application of the IP electric field through the Au lateral electrodes, considering the cantilever deflection. By considering the slope in Figure 5b, they estimated the IP piezoelectric coefficient, *e*_11_, of the monolayer MoS_2_ to be 2.9 × 10^−10^ C m^−1^. However, the IP piezoelectric coefficient obtained by this approach can include flexoelectric contributions that can be induced by the curvature of the sample and tip indentation. Thus, although the flexoelectric contribution was more than one order of magnitude lower than the piezoelectric contribution in their work (Figure 5a,b), the intrinsic piezoelectric coefficient may be significantly different if the flexoelectric contributions are significantly large.

Recently, Gomez et al. reported the direct detection of the piezoelectric coefficient by SPM‐based current detection with force modulation, called direct piezoelectric force microscopy (DPFM) (Figure 5c).^[^ ^164^ ^]^ When measuring the *F*–*D* curve, the piezoelectric charge, whose sign depends on the domain direction, can be generated by the force applied by the tip, as shown in Figure 5d. By obtaining the average of the generated current, the piezoelectric coefficient can be evaluated. Figure 5e,f shows the experimental results of piezoelectric charge generation in PPLN with the *F*–*D* spectroscopy mode. As the force/time rate increased, the obtained current increased because the piezoelectric charge decreased with time. Figure 5f shows the current collected in the different domain directions during the approach/retraction of the *F*–*D* spectroscopy measurements. It is noted that although the measurement of piezoelectric charge via DPFM is physically straightforward, it requires a low instrumental noise setup considering the generated current level (≈fA).

### 4.2 Evaluation of Ferroelectricity

In Section 3.2., we discussed PFM‐based approaches for evaluating ferroelectricity at the nanoscale. In these cases, ferroelectric domain switching was measured by monitoring the electric field‐induced surface displacement based on the converse piezoelectric effect. However, a typical method to evaluate ferroelectricity macroscopically involves *P*–*E* hysteresis loop measurements, whereby the ferroelectric polarization charge is measured by monitoring the switching current, rather than the electromechanical response. Unlike macroscopic ones, *P*–*E* hysteresis loop measurements based on SPM can be highly challenging owing to the extremely small top electrode area or contact area of the conductive SPM tip. The contact area, which is typically ≈300 nm^2^, is excessively small to obtain measurable switching currents considering the level of polarization charge density for classical ferroelectrics (50–100 µC cm^−2^)^[^ ^165^, ^166^ ^]^ and the influence of the parasitic capacitance from multiple sources.^[^ ^167^, ^168^, ^169^, ^170^ ^]^ For instance, the parasitic capacitance between the cantilever and sample surface can increase the instrumental noise level and eventually hinder the detection of the polarization charge through the SPM. Accordingly, there are some reports on methods to overcome these drawbacks or to understand the parasitic capacitance in SPM.^[^ ^167^, ^168^, ^169^, ^170^ ^]^

**Figure 6:**
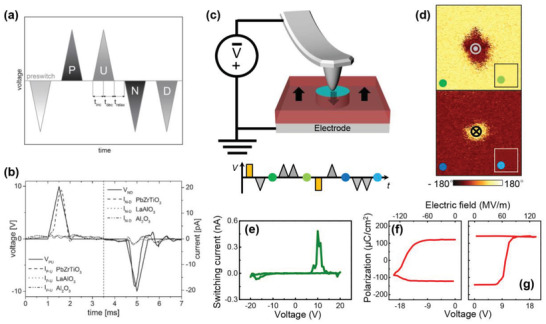
a) Waveform of the PUND method and b) corresponding current characteristics in PZT, LaAlO_3_, and Al_2_O_3_. (a) and (b) were reproduced with permission.^[^ ^37^ ^]^ Copyright 2017, AIP Publishing LLC. c) Schematic of AFM‐PUND measurement. d) PFM images before and after (inset) application of the AFM‐PUND waveforms. The colored circles in Figure (c) and (d) indicate the sequences in the PFM measurements. e) Switching current and f,g) corresponding *P*–*E* hysteresis loops on the negative (f) and positive (g) voltage sides, respectively. (c–g) were reproduced with permission.^[^ ^38^ ^]^ Copyright 2018, Wiley‐VCH Verlag GmbH & Co. KGaA.

Martin et al. suggested a new approach based on conductive atomic force microscopy (CAFM) combined with the positive‐up‐negative‐down (PUND) method.^[^ ^37^ ^]^ To obtain more accurate polarization charge values, they used the PUND waveform, as shown in **Figure** **6**a,^[^ ^11^, ^12^, ^14^, ^37^ ^]^ when a unipolar waveform is applied two times in the same direction, the switching current part is conveniently obtained. In other words, because switching is achieved by the first waveform, the switching current does not generate in the second waveform. In a similar manner, a ferroelectric polarization charge can be obtained by *I*–*V* curves through the PUND method in CAFM. The obtained switching current is shown in Figure 6b. In the case of LaAlO_3_ and Al_2_O_3_, which are non‐ferroelectric materials, it is possible to confirm that the current response hardly appears according to the applied waveform. However, the current response of PZT depends strongly on the applied voltage.

Kwon et al. suggested a similar approach to obtain the actual switched area by further combination with PFM, which is referred to as AFM‐PUND.^[^ ^38^ ^]^ In their work, they focused on the switched area rather than the contact area because the amount of polarization charge is eventually determined by the switched area in ferroelectric materials. In this case, quantitative evaluation of polarization charge density is possible because the switched area is measurable by PFM. If the switched area is sufficiently large, the polarization charge can be observed in the obtained current regardless of the contact area. The voltage waveform for the AFM‐PUND method is shown in the inset of Figure 6c, and the obtained switching current is presented in Figure 6e. Because the switched area is not confined in the AFM‐PUND method (unlike in the macroscopic *P*–*E* hysteresis loop measurement where it can be confined within the top electrode), the switched area needs to be determined via PFM images, as shown in Figure 6d. Based on the obtained switching current and switched area, nanoscale *P*–*E* hysteresis loops can be constructed, as shown in Figure 6f,g. We expect that this approach can be applied to measure a significantly smaller polarization charge density if the frequency of the waveform is higher than that (e.g., 1–16 Hz) used in the study. Nevertheless, further verifications based on the high‐frequency measurements are required.

**Figure 7:**
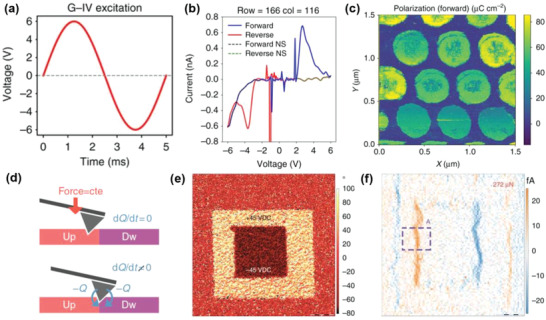
a) Schematic of the G‐IV waveform and corresponding b) *I*–*V* curve and c) spatial map of the polarization charge density. (a–c) were reproduced with permission.^[^ ^39^ ^]^ Copyright 2018, Springer Customer Service Center GmbH: Springer Nature. d) When the tip crosses different domains, a current flows as the force is kept constant; however, the sign of the *d_33_* value is inverted. e) PFM phase image of a 400 nm‐thick BFO film, where two opposite domains were previously generated. f) DPFM image for the scan direction from left to right. (d–f) were reproduced with permission.^[^ ^164^ ^]^ Copyright 2017, Springer Customer Service Center GmbH: Springer Nature.

Similar to the AFM‐PUND approach, Somnath et al.^[^ ^39^ ^]^ determined the polarization charge in a PZT nanocapacitor based on current detection. As described above, when *I*–*V* curves are measured by SPM, the switching current is unlikely to be detected owing to the small switching current compared with the parasitic capacitance. To increase the switching current, Somnath et al.^[^ ^39^ ^]^ obtained *I*–*V* curves were by applying a high‐frequency voltage waveform (200 Hz) in the PZT nanocapacitor, as shown in **Figure** **7**a. After the current contribution from the capacitance was removed by Bayesian inference, switching current peaks were obtained (Figure 7b). The approach, which is referred to as general mode *I*–*V* (G‐IV), allows the spatial visualization of the polarization charge density, as shown in Figure 7c.

DPFM can also be used to evaluate ferroelectricity.^[^ ^164^ ^]^ When the SPM tip crosses the domain wall, current flows under the constant force (Figure 7d). Thus, after the poling procedure of box patterns, similar to Figure 5e,f, piezoelectric charge generation occurs in the domain walls and can, therefore, be detected by DPFM instead of conventional PFM. In this approach, because different signs of the piezoelectric charges can be generated and detected depending on the domain, for example, polarization alignment, it can be used for evaluating ferroelectricity as well. Figure 7e,f shows the PFM phase and the corresponding DPFM images for the box patterns in a BFO thin film. The DPFM image shows the current flowing at the domain walls.

**Figure 8:**
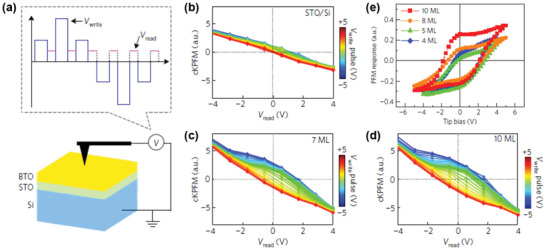
a) Schematic of the cKPFM experimental setup. cKPFM curves measured after application of different write voltage pulses for b) the STO/Si stack, c) 7 ML‐thick, and d) 10 ML‐thick BTO films. e) PFM hysteresis loops for BTO film thicknesses of 4–10 ML. (a–e) were reproduced with permission.^[^ ^177^ ^]^ Copyright 2017, Springer Customer Service Center GmbH: Springer Nature.

Few studies have attempted to evaluate ferroelectricity using Kelvin probe force microscopy (KPFM) or electrostatic force microscopy (EFM). However, both KPFM and EFM provide information on surface potential through the summation of all the surface charges rather than only the ferroelectric polarization charges.^[^ ^171^, ^172^ ^]^ In particular, because the application of an electric field through the SPM tip can induce water dissociation and/or charge injection, the origin of the surface potential can be significantly more complicated.^[^ ^172^, ^173^, ^174^, ^175^, ^176^ ^]^ Thus, the measurement of ferroelectric properties using these SPM techniques is not suitable for establishing the presence of ferroelectricity. Nonetheless, contact KPFM (cKPFM) can provide some insight into ferroelectricity because of the different operational principle compared with that of typical KPFM. Yang et al.^[^ ^177^ ^]^ reported that cKPFM can also be used to distinguish the ferroelectricity of materials by observing the two‐remnant offset behavior, which is not exhibited by non‐ferroelectric materials, as shown in **Figure** **8**. Figure 8a schematically shows the cKPFM experimental setup that applies multiple cycles of DC pulses (*V*_write_) and subsequently varies the DC pulse with AC voltage to detect the junction potential (*V*_read_).^[^ ^90^ ^]^ The cKPFM measurements revealed that two distinct remnant offsets at 0 *V*_read_ were not observed in the non‐ferroelectric SrTiO_3_/Si stack, while they were clearly observed in the ferroelectric BaTiO_3_ thin films (Figure 8b–d). This behavior reveals the discrimination of the ferroelectric state. However, similar to the non‐piezoelectric contributions in PFM, there might also be undesired contributions to cKPFM signals that have not yet been fully studied. This indicates that careful interpretation of the signals and further studies are necessary to deeply understand ferroelectricity in cKPFM, as well as in other SPM‐based techniques.

## 5 Summary and Outlook

We discussed the SPM‐based techniques used for evaluating the piezoelectric and ferroelectric properties at the nanoscale. We addressed the recent advances in PFM and some of its issues, including quantification and non‐piezoelectric effects. Then, other SPM‐based techniques were briefly introduced, for example, the CAFM technique, which has been used to evaluate the piezoelectric coefficient by measuring the charge generated by the direct piezoelectric effect. We also reported the evaluation of ferroelectricity through the direct measurement of the switching current using AFM‐PUND and high‐frequency current acquisition using G‐IV.

PFM is becoming one of the well‐established tools for exploring nanoscale piezoelectric and ferroelectric phenomena. However, as the application fields of PFM expand, limitations on the exact data measurement and interpretation have also arisen. Moreover, although the CAFM and cKPFM approaches for evaluating piezoelectric and ferroelectric properties have been suggested, their applicability should be further demonstrated in diverse material systems. Thus, continuous development and improvement of the SPM‐based techniques are necessary for effectively investigating piezoelectric and ferroelectric properties. For instance, SPM‐based techniques could be combined with other chemical microscopic tools to facilitate both the physical and chemical understanding of nanoferroic phenomena.^[^ ^131^, ^178^ ^]^ Further, multiple SPM‐based techniques could be combined for evaluating piezoelectric and ferroelectric properties accurately. We anticipate that, based on this progress, SPM can become a comprehensive platform for the evaluation of piezoelectric and ferroelectric properties at the nanoscale.

## Conflict of Interest

The authors declare no conflict of interest.
